# Episodic Eruptions of Volcanic Ash Trigger a Reversible Cascade of Nuisance Species Outbreaks in Pristine Coral Habitats

**DOI:** 10.1371/journal.pone.0046639

**Published:** 2012-10-04

**Authors:** Tom Schils

**Affiliations:** University of Guam Marine Laboratory, UOG Station, Mangilao, Guam, United States of America; Victoria University Wellington, New Zealand

## Abstract

Volcanically active islands abound in the tropical Pacific and harbor complex coral communities. Whereas lava streams and deep ash deposits are well-known to devastate coral communities through burial and smothering, little is known about the effect of moderate amounts of small particulate ash deposits on reef communities. Volcanic ash contains a diversity of chemical compounds that can induce nutrient enrichments triggering changes in benthic composition. Two independently collected data sets on the marine benthos of the pristine and remote reefs around Pagan Island, Northern Mariana Islands, reveal a sudden critical transition to cyanobacteria-dominated communities in 2009–2010, which coincides with a period of continuous volcanic ash eruptions. Concurrently, localized outbreaks of the coral-killing cyanobacteriosponge *Terpios hoshinota* displayed a remarkable symbiosis with filamentous cyanobacteria, which supported the rapid overgrowth of massive coral colonies and allowed the sponge to colonize substrate types from which it has not been documented before. The chemical composition of tephra from Pagan indicates that the outbreak of nuisance species on its reefs might represent an early succession stage of iron enrichment (a.k.a. “black reefs”) similar to that caused by anthropogenic debris like ship wrecks or natural events like particulate deposition from wildfire smoke plumes or desert dust storms. Once Pagan's volcanic activity ceased in 2011, the cyanobacterial bloom disappeared. Another group of well-known nuisance algae in the tropical Pacific, the pelagophytes, did not reach bloom densities during this period of ash eruptions but new species records for the Northern Mariana Islands were documented. These field observations indicate that the study of population dynamics of pristine coral communities can advance our understanding of the resilience of tropical reef systems to natural and anthropogenic disturbances.

## Introduction

Critical transitions in tropical reef systems are often characterized by a shift from a coral-dominated state to a macroalgal-dominated system [1], which is mediated by a change in the microbial communities of the coral holobiont [2]. The coral-algal phase shift paradigm was developed for Caribbean reefs, which have undergone dramatic transitions to algal-dominated states [3]. The extent and degree of such critical transitions in the Pacific are less clear [4], [5]. Blooms of cyanobacteria are also regarded to be a sign of reef degradation and are becoming more frequent in the Pacific [6], Caribbean [7], and the Atlantic [8], [9]. The increased global environmental impact of cyanobacterial blooms on tropical reef systems has been attributed to their tolerance of strong solar radiation, higher temperatures, and abundant nutrients [10]. Positive feedback mechanisms like a decrease in coral recruitment [6], a reduction in coral growth [11], or the induction of lethal coral diseases [12] have a long-term effect on the recovery of disturbed coral communities. Other environmental impacts of cyanobacterial blooms include fish die-offs [13] and human health risks [14]. A better understanding of the temporal dynamics and environmental impact of cyanobacterial blooms in coastal environments are required to develop management strategies for coastal ecosystems where the abundance of cyanobacteria is on the rise.

A recent study reported on the effects of volcanic ash deposits on the benthic communities and the reef fish populations of Anatahan Island in the Mariana Arc [15]. Since pyroclastic deposits were high during the 2003 eruption of Anatahan, the benthic assemblages of the island were mainly affected by ash burial and smothering resulting in a low cover of live coral, crustose coralline algae and other macroalgae. Similar observations of a decline in the cover of all benthic biota due to ash and scoria smothering, scouring and burial were made for Pagan Island, Mariana Islands, shortly after its large eruption in 1981 [16], [17]. Besides the impacts of ash burial [15], [18], no additional findings on the effects of moderate volcanic ash deposition on (sub)tropical Pacific reefs have been published. This lack of information prevents impact assessments and comparisons of such disturbances in the region as concluded in the paper describing the effects of the 1981 eruption of Mount Pagan [17]: “a thorough literature search provided no published information on the ecological impacts of ash fall”. Recent benthic surveys in Pagan, about 185 km north of Anatahan, reveal new insights on the effect of an episodic phase of low-level ash and gas eruptions on benthic assemblages of pristine tropical reef systems.

## Methods

### Site description

Pagan is the largest island of the northern Mariana Arc (48 km^2^) and the fifth largest of all Mariana Islands [19]. Pagan lacks a limestone cap as found in the southern Mariana Islands. Although Holocene reef deposits are present, biogenic carbonate deposition is more limited than in the southern Mariana Islands and certain stretches of coastline are devoid of any surfacing Holocene reef which is buried under recent volcanic deposits. The dramatic impact of natural disturbances shapes the coral communities of the northern Mariana Islands, which are less diverse than those of the southern islands [20] and their coral surface area is negatively correlated with latitude and island age [21]. Population density of corals, however, does not differ between the northern and southern Mariana Islands, indicating that the connectivity of recruits between and within these islands is not a limiting factor for coral reef development [20].

**Figure 1:**
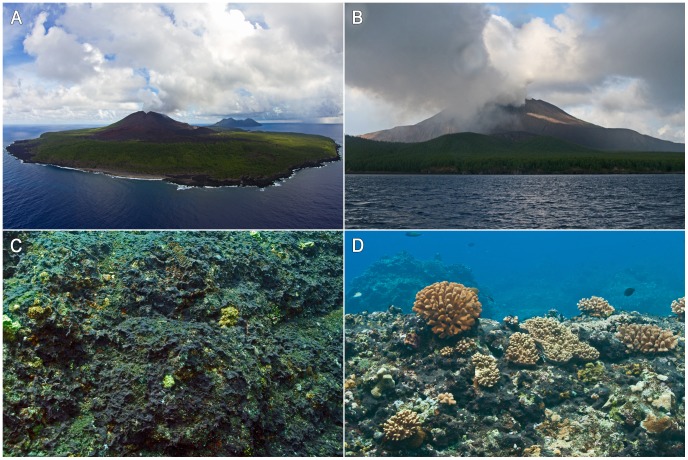
Pagan and its reef assemblages. A. Northeast to southwest view of Pagan Island. B. Ash and gas plume emitted from Mount Pagan on July 14, 2010. C,D. High cyanobacterial cover on hard-bottom reef habitats.

Pagan Island is shaped around a northern and a southern volcano connected by a narrow isthmus (Fig. 1A), with the northern volcano being the most active from at least the 17^th^ century onwards [22]. The largest eruption of Pagan in recent history occurred in 1981 [23] and instigated the evacuation of its fifty-some permanent residents. Since then, few temporary residents have lived on the island with lately just one or two official representatives of the mayor of the Northern Mariana Islands. The strong volcanic activity in 1981 was followed by intermittent light ejection of primarily phreatic ash until 1996 [24]. From 1996 onwards no emissions were documented until early December 2006 when ash and gas plumes were reported by local residents and were observed in satellite imagery [25]. This stint of eruptive activity ended later that month, and the volcano remained quiet until mid-April 2009. Although ground-based geophysical instruments are lacking on Pagan, USGS records show that volcanic activity increased in 2009 and frequent observations of gas and ash emissions have been documented up to December 2010 (Fig. 1B).

### Survey methods

Benthic cover estimates for Pagan Island were collected during a natural resource assessment study in 2010 [26] (hereafter USFWS dataset) and obtained from a Pacific-wide monitoring program [19] (hereafter MARAMP dataset). All benthic surveys of the USFWS dataset were conducted by the author. Percent cover estimates of algae, corals, and other non-scleractinian invertebrates were assessed *in situ*, mostly to species level. Within a site, six 0.25 m^2^ quadrats were surveyed along a 25 m benthic transect line. Similar benthic cover data for Guam and Tinian collected by the same observer were used to compare cyanobacterial cover between islands. Percent cover estimates of the MARAMP dataset were based on the line point intercept method with a sampling interval of half a meter and this along two 25 m transects per site. These surveys were conducted biennially from 2007 onwards by staff of the Coral Reef Ecosystem Division (NOAA).

### Analysis of survey data

Due to the random sampling design, a substantial proportion of the surveys were conducted over bare substratum, which consisted predominantly of lava sand. Hence, the comparison of cyanobacterial cover between islands was based on transect data with a total biotic cover greater than 30%. Next, the data from both the USFWS and the MARAMP datasets were analyzed alike as in both cases the untransformed data (i) were not normally distributed and (ii) did not have homogeneous variances across the different groups. First, the optimal lambda for a power transformation was calculated (rounded to the closest multiple of 0.5) and the data was Box-Cox transformed. The approximate lambda was −0.5 in both cases, representing a reciprocal square root transformation. The transformed data achieved the assumption of homogeneity of variances, but not normality. Then, non-parametric one-way analyses of variance with post-hoc tests based on 9,999 permutations were run on the transformed data [27], [28]. Box plots were generated using the untransformed data while the significance tests were based on the transformed data. All analyses were conducted in the R statistical package [29].

## Results and Discussion

### Cyanobacterial bloom

**Figure 2:**
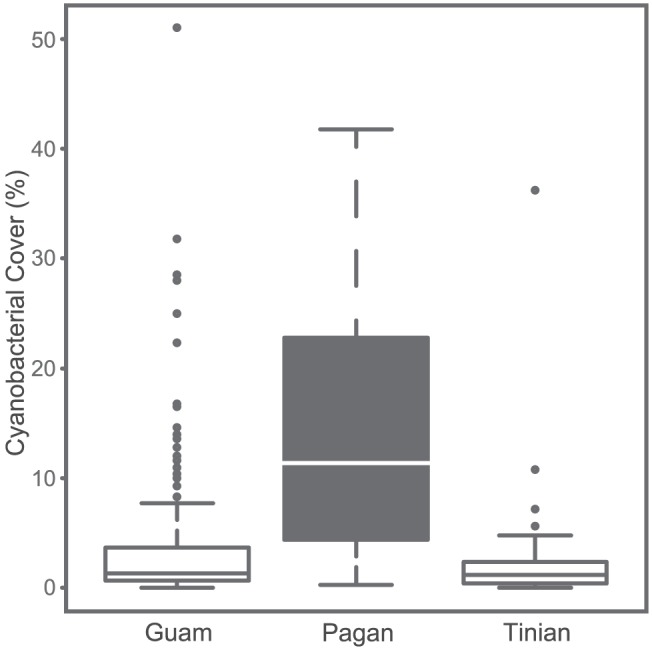
Cyanobacterial cover for 222 transects of three islands within the Mariana Arc. Significant differences between islands (*P*<0.001) are indicated by boxes of contrasting shades. The median cyanobacterial cover per island is indicated by a horizontal line. The box represents the inter-quartile range (IQR) between the upper and lower quartile. Whiskers maximally extend 1.5 times beyond the IQR and outliers are indicated by circles. All transects were surveyed by a single observer based on visual estimates of percent cover in quadrats along transects. Pagan surveys were all conducted in 2010.

**Figure 3:**
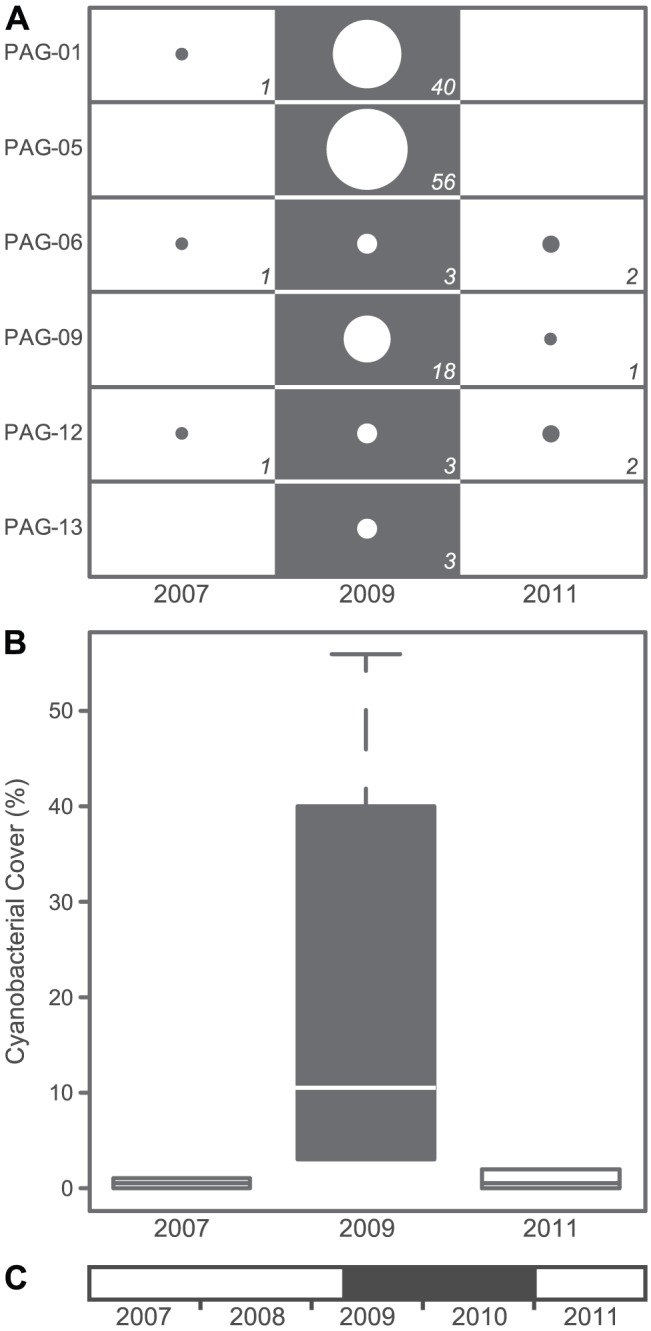
Cyanobacterial cover and volcanic activity in Pagan over the course of a five year period. A. Balloon plot of cyanobacterial cover per site in 2007, 2009, and 2011. Numbers represent percent cover when cyanobacteria were observed for the site. Each of the six sites was surveyed three times using the line point intercept method. Years with contrasting fill shades differed significantly (*P*<0.01). B. Box plot depicting cyanobacterial cover of all sites over a five year time span. Box plot specifications as in Fig. 2. C. Time line from 2007 to 2011 with the period of volcanic activity in grey.

A spatial comparison of benthic data from 222 survey sites showed a significant difference in cyanobacterial cover between the islands of Guam, Pagan, and Tinian in the Mariana Arc (F_2,219_ = 20.008, *P*<0.001; Fig. 2). Post-hoc comparisons indicated that the cyanobacterial cover of Pagan in 2010 (14.1±2.4%; mean ± SE) was significantly higher than that of the other two neighboring islands. Cyanobacterial cover did not differ significantly between Guam (3.8±0.5%) and Tinian (2.7±0.9%). Although cyanobacterial blooms have been reported for other islands in the Mariana Arc [6], they are generally localized and do not cover entire coastlines of these islands. A temporal comparison of cyanobacterial cover for permanent monitoring sites in Pagan (MARAMP dataset) also identified a sudden bloom of cyanobacteria in 2009 and a likewise sudden decline in 2011 (F_2,15_ = 14.206, *P*<0.001; Fig. 3A). While cyanobacterial cover in 2009 was significantly higher than before and after the bloom, post-hoc comparisons showed no significant difference in cyanobacterial cover between the surveys conducted in 2007 and 2011 (Fig. 3B). The 2009–2010 bloom of cyanobacteria coincides with the volcanic activity of Mount Pagan (Fig. 3C) that started in the month of April in 2009 and ash and gas plumes persisted until the end of 2010. The stark temporal dynamics in cyanobacterial cover from an average of 0.5±0.2% (mean ± SE) in 2007, to 20.5±9.2% in 2009, and back to 0.8±0.4% in 2011 are suggestive of a link between atmospheric ash deposits and the vigorous growth of cyanobacteria. *Blennothrix lyngbyacea* (Kützing ex Gomont) Anagnostidis & Komárek was the dominant species in the cyanobacterial assemblages of Pagan Island. Food composed of crude extracts from a mixed cyanobacterium assemblage from Guam that included *B. lyngbyaceus* was significantly avoided by grazing parrotfish [13]. Population outbreaks of this species in Puerto Rico could not be linked to differences in water temperature or nitrate and nitrite concentrations [7] and some of the proposed contributing factors of localized blooms in Guam include low wave action, increased phosphate levels, and augmented iron bioavailability [6], [30], [31].

A blue tint in the false-color satellite image of June 3, 2010, suggests that the ash plume was enriched in sulfate aerosols [25]. Sulfur smells were also documented during the eruptions of 1981 and 2006. Blooms of benthic cyanobacteria have been reported for shallow coastal hydrothermal vents of the volcanically active Kermadec Islands, which are rich in sulfur compounds [32]. In such ecosystems, cyanobacteria use the energy of reduced compounds in volcanic effluents for autotrophic production of new organic matter, hereby increasing their competitive advantage over other benthic and planktonic biota resulting in blooms. However, shallow hydrothermal vents of the nearby sunken caldera of Maug which chronically release reduced sulfur compounds are not characterized by high cyanobacterial cover but are carpeted by brown and green macroalgae. Furthermore, the gas hydrogen sulfide of volcanic smoke plumes will not precipitate locally in large concentrations, making it an unlikely causal factor of the bloom. Pagan's volcanic rocks mainly consist of silicon dioxide (SiO_2_, 50.5%), aluminum oxide (Al_2_O_3_, 17.4%), and iron oxides (Fe_2_O_3_ and FeO, 11.2%) [33], [34]. The latter compounds are the most likely candidates to stimulate cyanobacterial growth as iron requirements of cyanobacteria are greater than other algal groups because of their high PS I to PS II ratio [35] and the presence of nitrogenase in diazotrophs [36]. The iron limitation of cyanobacteria in oligotrophic coral habitats has been attributed to their evolutionary origin in an anoxic ocean with high bioavailabilty of iron [36]. When volcanic ash dissolves in seawater bioactive trace metals like iron become immediately available to primary producers [37] and experimental studies have demonstrated that this increase in iron concentration can initiate cyanobacterial blooms [38]. Unfortunately, nutrient data of the coastal waters off Pagan were not available for the years 2007–2011.

### Terpios

At three sites along the western side of Pagan, large patches of *Terpios hoshinota*, a sponge notorious for killing and overgrowing large patches of live coral colonies, were observed to expand simultaneously with the cyanobacterial bloom. In contrast to the statistical analysis of cyanobacterial cover, a spatial comparison of *Terpios* cover between islands or a temporal analysis of *Terpios* outbreaks on Pagan reefs was not possible because (i) the MARAMP protocol did not include sponge cover as a monitoring parameter and (ii) *Terpios* outbreaks in the region are rather infrequent and highly localized. The latter observation is consistent with the long-term dynamics of *Terpios* colonies in the Ryukyu Islands where prominent, yet infrequent, outbreaks had a large impact on the benthic reef communities but allowed for recovery [39]. The *Terpios* colonies in Pagan, however, exhibited three opportunistic growth characteristics concurrent with the cyanobacterial bloom and the increased volcanic activity: (i) its facultative symbiosis with multicellular cyanobacteria, (ii) altered substrate specificity, and (iii) increased growth speed.

**Figure 4:**
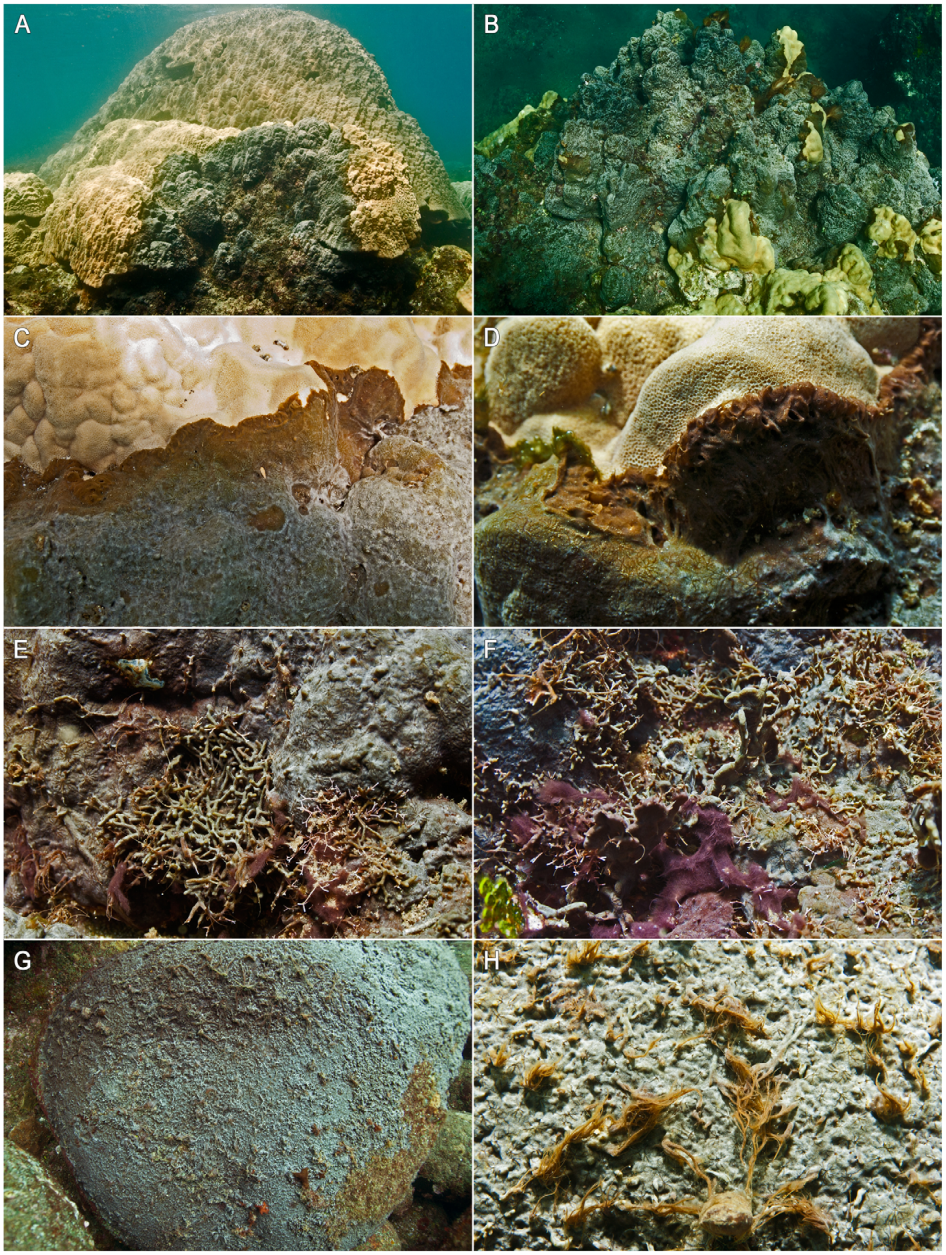
*Terpios* and associated cyanobacteria overgrowing corals, macroalgae, and bare reef substrates. A,B. *Terpios* overgrowing some of the largest *Porites* colonies found in Pagan. C,D. Vigorous outgrowths of filamentous cyanobacteria at the coral-facing margin of the sponge. E,F. Fleshy macroalgae [*Gracilaria salicornia* (C. Agardh) E.Y. Dawson], calcified green algae (*Halimeda* spp.), and geniculate coralline algae (*Amphiroa* and *Jania* spp.) overgrown by *Terpios*. G. *Terpios* outbreak covering large areas of otherwise turf or biofilm covered boulders. H. Close-up of *Terpios* overgrowing boulders with tufts of filamentous cyanobacteria emerging from the sponge surface.

Illustrating the origin of the vernacular name “killer sponge”, *Terpios* colonies were observed to actively overgrow massive *Porites* colonies as reported for other islands in the Mariana Arc [40]. Some of the largest and centuries-old *Porites* colonies (Figs 4A–D) of Pagan in Bandeera Bay were particularly affected by *Terpios* infestations. Large macroalgae were also draped with sponge tissue and their intact upright habit served as a testament of the speed by which *Terpios* colonies were progressing (Figs 4E–F). The sponge is known to grow on compact *Halimeda* clumps or on the base of larger *Halimeda* thalli and on non-geniculate algae, but not on geniculate (articulated) algae [41], [42]. During the cyanobacterial bloom in Pagan, however, *Terpios* completely overgrew erect *Halimeda* thalli and geniculate coralline algae of the genera *Amphiroa* and *Jania* (Figs 4E–F). Besides overgrowing sessile macrobenthos, this is the first report of the sponge blanketing large areas of volcanic boulders, which were initially covered by a thin layer of algal turf or microbial biofilm (Figs 4G–H). Filamentous strands of cyanobacteria were growing vigorously out of the sponge tissue. When overgrowing and competing with sessile biota, the filamentous cyanobacteria were densely concentrated at the growth margin of the sponge colony. Hence, the presence of filamentous cyanobacteria in the sponge tissue seems to instigate a competitive advantage for *Terpios* when expanding over the surrounding benthos. A detailed assessment of this symbiotic relationship requires further experimental research. Thus far, *Dysidea granulosa* and *Lamellodysidea herbacea* were the only sponges of the Mariana Islands for which a symbiosis with a filamentous cyanobacterium (*Oscillatoria* cf. *spongeliae*) has been reported [43], [44]. Of all sponge/cyanobacterium symbioses, those with filamentous cyanobacteria constitute a minority. *Terpios hoshinota* contains symbiotic unicellular cyanobacteria, dominated by an unidentified species related to representatives of the genus *Prochloron* [45] and these unicellular symbionts are vertically transferred through *Terpios* larvae [46]. This presumably obligate symbiosis has been targeted in management actions to halt *Terpios* outbreaks and to kill the sponge by shading [47]. The bacterial communities of *Terpios*-affected corals include coral pathogens which are assumed to benefit the sponge's interactions with other benthic biota especially in the regions closest to the margin of the sponge [45]. The large tufts of filamentous cyanobacteria that were especially abundant in the *Porites*-facing front of *Terpios* colonies (Fig. 4D), constitute a new but related sponge-coral interaction type. A comparative study of *Terpios* interactions with 20 coral species revealed no species-specific morphological interaction traits [48], indicating that this new interaction mode supported by a symbiosis with filamentous cyanobacteria could result from the particular environmental conditions created by volcanic ash deposition. This would imply that *Terpios* outbreaks would mostly be a threat in disturbed environments characterized by an abundance of (pathogenic) bacteria.

### Pelagophyceae

Algae of the class Pelagophyceae are another group of sessile benthic organisms that manifest rapid outbreaks on Pacific reefs [49]. Three species of “golden algae” were observed during the surveys: *Chrysocystis fragilis* Lobban, Honda & Chihara, *Chrysonephos lewisii* (Taylor) Taylor, and *Sarcinochrysis marina* Geitler. The latter two constitute new species records for Pagan and the northern islands of the Mariana Arc (*i.e.*, the islands north of Saipan). Pelagophyceae were infrequently encountered and always at low cover densities. The absence of pelagophyte blooms on Pagan's reefs during the period of ash eruptions is consistent with the observation that elevated temperatures in oligotrophic waters of the Great Barrier Reef trigger *C. fragilis* blooms on recently killed coral colonies following crown-of-thorns starfish outbreaks or extensive bleaching events [49], [50]. Thus, the naturally low coral cover on Pagan's reefs might restrict the frequency and extent of pelagophyte blooms on Pagan's reefs.

### Critical transitions

The here-presented field observations suggest a relationship between the episodic volcanic ash eruptions of Mount Pagan and a sudden cyanobacterial bloom on its pristine reefs. This would imply that iron enrichments in coastal reefs not only affect central Pacific coral atolls lacking exposed igneous rocks [51] but can also impact reef communities of volcanic islands that are not completely covered by carbonate platforms. During the cyanobacterial bloom, the newly documented symbiosis between filamentous cyanobacteria and *Terpios* was associated with rapid sponge growth and the aggressive overgrowth of coral colonies and substrate types hitherto unknown to be colonized by the sponge. Conclusive evidence of such relationships, however, can only be obtained through experimental studies. The rapid reversal of Pagan's cyanobacteria-dominated reefs to a state of low cyanobacterial cover once the episodic volcanic activity ceased demonstrates the resilience of these remote pristine reefs. Whereas the resurgence of volcanic ash deposits corresponded to a bloom of cyanobacteria, regionally important nuisance species of the Pelagophyceae did not reach bloom densities. It is likely that trace elements in the volcanic deposits, particularly iron, spurred cyanobacterial growth and that the pre-reversal cyanobacterial bloom represented an early succession stage of reef degradation leading to less diverse benthic communities [52]. These observations demonstrate that remote island systems are living laboratories which can reveal new ecological phenomena, elucidate natural dynamics of reef systems, and guide experimental research on disturbances impacting reef health.
